# Sugammadex use in pediatric patients with stage IV-V chronic kidney disease in a quaternary referral hospital: a case series

**DOI:** 10.1186/s12871-024-02584-9

**Published:** 2024-06-10

**Authors:** Sindhu N. Samba, Youssef Daklallah, Sydney E. S. Brown, Douglas A. Colquhoun, Zubin J. Modi, Rebecca Nause-Osthoff

**Affiliations:** 1 Department of Anesthesiology, Pediatric Division, C.S. Mott Children’s Hospital, University of Michigan, 4-911 Mott Hospital / 1540 E. Hospital Dr, SPC 4245, Ann Arbor, MI 48109 USA; 2 Department of Anesthesiology, University of Michigan, Ann Arbor, MI USA; 3 Division of Pediatric Nephrology, Department of Pediatrics, University of Michigan, Ann Arbor, MI USA; 4 Susan B. Meister Child Health Evaluation and Research (CHEAR) Center, Department of Pediatrics, University of Michigan, Ann Arbor, USA

**Keywords:** Sugammadex, Chronic kidney disease, Renal impairment, Pediatric, Neuromuscular blockade, Recurarization, Outcome

## Abstract

**Background:**

Sugammadex is a pharmacologic agent that provides rapid reversal of neuromuscular blockade via encapsulation of the neuromuscular blocking agent (NMBA). The sugammadex-NMBA complex is primarily cleared through glomerular filtration from the kidney, raising the possibility that alterations in renal function could affect its elimination. In pediatric patients, the benefits of sugammadex have led to widespread utilization; however, there is limited information on its application in pediatric renal impairment. This study examined sugammadex use and postoperative outcomes in pediatric patients with severe chronic renal impairment at our quaternary pediatric referral hospital.

**Methods:**

After IRB approval, we performed a retrospective analysis in pediatric patients with stage IV and V chronic kidney disease who received sugammadex from January 2017 to March 2022. Postoperative outcomes studied included new or increased respiratory requirement, unplanned intensive care unit (ICU) admission, postoperative pneumonia, anaphylaxis, and death within 48 h postoperatively, unplanned deferral of intraoperative extubation, and repeat administrations of NMBA reversal after leaving the operating room.

**Results:**

The final cohort included 17 patients ranging from 8 months to 16 years old. One patient required new postoperative noninvasive ventilation on postoperative day 2, which was credited to hypervolemia. Another patient had bronchospasm intraoperatively resolving with medication, which could not definitively be associated sugammadex administration. There were no instances of deferred extubation, unplanned ICU or need for supplemental oxygen after tracheal extubation identified.

**Conclusion:**

No adverse effects directly attributable to sugammadex in pediatric patients with severe renal impairment were detected. There may be a role for utilization of sugammadex for neuromuscular reversal in this population.

## Background

Sugammadex is a modified γ-cyclodextrin that encapsulates and inactivates the steroidal non-depolarizing neuromuscular blocking agents (NMBAs), rocuronium and vecuronium. In patients with normal renal function, the NMBA-sugammadex complex is predominantly excreted in the urine. However, severe renal impairment may lead to decreased clearance and potential disassociation of the NMBA-sugammadex complex with redistribution of unbound NMBA from peripheral tissue compartment to central and effect site compartments. Consequently, a theoretical concern exists for recurarization resulting in adverse effects such as hypoxia, aspiration, or respiratory compromise including need for airway intervention. Both sugammadex and the NMBA-sugammadex complex can be effectively removed via high-flux hemodialysis but the use of sugammadex in patients with a glomerular filtration rate (GFR) of less than 30 ml/min, including those requiring dialysis, is not recommended by the manufacturer [1, 2].

Muscle relaxation in renally impaired patients can be achieved with cisatracurium via its unique mechanism of Hoffman elimination, which is independent of renal and liver function. An alternative muscle relaxant such as rocuronium may be indicated in the setting of renal impairment in some circumstances such as an allergy to cisatracurium or when a rapid sequence induction is indicated. Currently, there is minimal guidance on clinical practice in these scenarios. Despite manufacturer recommendations, there may be clinical situations in which administration of sugammadex in a pediatric patient with severe renal impairment may be clinically preferable to alternatives. A randomized double-blinded study in adult patients with GFR less than 30 ml/min found that return of train-of-four ratio to ≥ 90% after reversal of rocuronium with sugammadex was significantly faster compared to reversal of cisatracurium with neostigmine and glycopyrrolate (3.5 min vs 14.8 min, *P* < 0.0001) [3]. Reversal with sugammadex also results in lower rates of residual neuromuscular blockade and decreased postoperative pulmonary complications in adult patients relative to its predecessor, neostigmine [4–6]. Similarly, some pediatric patients with severe renal impairment at risk for postoperative pulmonary complications or other sequalae of incomplete reversal, may benefit from its use. Additionally, inadvertent administration of steroidal NMBAs may occasionally occur in patients with severe renal impairment, and clinicians may find themselves weighing the benefits of earlier reversal with sugammadex versus delaying reversal until shallower degree of neuromuscular block is attained when neostigmine may be utilized. Data to guide decision-making in these scenarios is lacking. Studies in adults with end stage kidney disease (ESKD) receiving sugammadex found evidence of postoperative hypoxemia and reintubation but no documented recurarization [7, 8]. The literature in pediatric patients with renal impairment receiving sugammadex includes only a handful of case reports [9, 10]. Therefore, we aimed to identify these patients and carefully study intraoperative and postoperative events surrounding its administration.

## Methods

This is a retrospective single center observational study at a quaternary referral university hospital. Institutional Review Board (IRB) approval was obtained with written consent waived by the University of Michigan IRB (IRBMED: HUM00216728). This article adheres to the applicable Enhancing the QUAlity and Transparency Of health Research (EQUATOR) and Case Reports (CARE) guidelines. An eligible cohort was identified using data from the Research Data Warehouse at University of Michigan. Children ages 0–17 years with stage IV (estimated glomerular filtration rate (eGFR) 15–29) or stage V (eGFR < 15) chronic kidney disease (CKD) receiving intraoperative sugammadex at C. S. Mott Children’s Hospital from 1/1/2017 to 3/15/2022 were included. Patients with significant renal impairment were identified using International Classification of Diseases (ICD)-10 codes and their electronic medical records were manually reviewed for diagnosis of stage IV or V CKD. To meet criteria for CKD, CKiD U25 formula was applied using serum creatinine labs drawn from day of surgery and three months prior [11]. Four patients did not have serum creatinine lab values three months prior but were included after manual chart review was consistent with clear diagnosis of CKD stage IV or V at the time of surgery. Patients with normal renal function or CKD stages I, II, or III as well as patients intubated prior to induction of anesthesia or with planned postoperative intubation were excluded. Electronic query was utilized to obtain patient age, sex, ASA classification, ideal and actual body weight and procedure associated with the anesthetic. Manual chart review of each patient was performed to obtain patient comorbidities, rocuronium and sugammadex dosing, last documented train of four (TOF), TOF after sugammadex, neostigmine dosing (if administered in addition to sugammadex), preoperative respiratory support, and preoperative and postoperative dispositions. The following outcomes were also obtained: postoperative reintubation, noninvasive ventilation, new supplemental oxygen, unplanned ICU admission, postoperative pneumonia, anaphylaxis, and death within 48 h postoperatively, unplanned deferral of intraoperative extubation, and repeat administrations of NMBA reversal after leaving the operating room. Three independent anesthesiologist reviewers evaluated outcomes; discrepancies were adjudicated until consensus was reached.

## Results

**Fig. 1:**
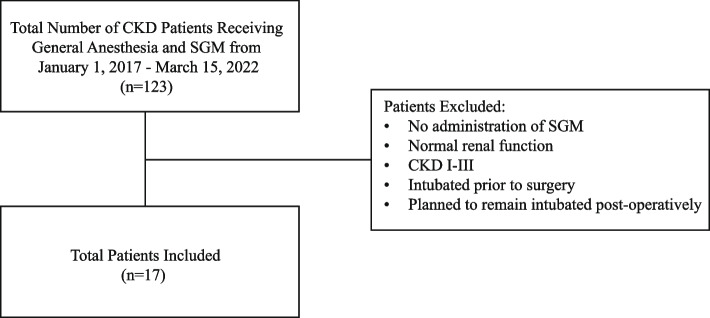
Study flowchart. CKD = chronic kidney disease. SGM = sugammadex

The initial cohort identified 123 patients. 106 patients were excluded because of 1) normal renal function at the time of sugammadex administration or chronic kidney disease stages I-III (*n* = 100), 2) no administration of NMBA and/or sugammadex (*n* = 1), 3) intubated prior to surgery (*n* = 2), and 4) plan to remain intubated postoperatively (*n* = 3). The final cohort had a total of 17 patients (Fig. 1). Patient age ranged from 8 months to 16 years old and 88% (*n* = 15) underwent their procedures during daytime, weekday hours. Four patients underwent renal transplant; the remainder underwent a variety of other procedures (Table 1). Patients received between 1.85–4.34 mg/kg of sugammadex based on last documented TOF. TOF was not documented in two cases. One patient received less than the recommended dose (2 mg/kg given for 0/4 twitches). This patient utilized preoperative respiratory support (noninvasive ventilation, BiPAP, nightly at home) and did not require escalated support postoperatively, despite the reduced dose. Of the remaining patients, one (1/16) required new postoperative non-invasive ventilation (BiPAP on postoperative day 2); however, contemporaneous clinical documentation ascribes this to be secondary to hypervolemia rather than residual neuromuscular blockade. The patient had been extubated to room air and transported to pediatric ICU for post-transplant care. Hypotension and low urine output resulted in multiple crystalloid and albumin boluses with subsequent hypoxemia. BiPAP was utilized for one day and diuresis was initiated, after which the patient tolerated weans to nasal cannula and room air by POD5. Physical exam documented by the critical care physician demonstrated crackles at the bases to auscultation. Chest x-ray had evidence of interstitial and alveolar edema, moderate bilateral pleural effusions and cardiomegaly. The patient’s weight was also increased by 4.3 kg by POD2. An echo was obtained showing normal biventricular function, normal right ventricular systolic pressure (RVSP) and mildly dilated left atrium. No other patients required new supplemental oxygen, reintubation or deferred extubation (an unplanned delay to expected extubation time), and no patients received additional doses of sugammadex for reversal. There were no instances of unplanned ICU admission, postoperative pneumonia or evidence of worsening renal impairment in the postoperative period within 30 days.

**Table 1:** Summary of patient demographics and clinical characteristics of study cohort

CKD Stage	Age (years)	Dialysis (Y/N)/Type	Comorbidities	Procedure	Total Intraoperative Rocuronium (average mg/kg/hr)	Last Rocuronium Dose to Sugammadex (min)	Last Documented TOF	Sugammadex Dose (mg/kg)	Sugammadex to Extubation Time (min)	New Onset Respiratory Support	Anaphylaxis
V	9 months^a^	N	High output CKD, atrophic kidney, posterior urethral valve, neurogenic bladder, myelomeningocele s/p VP shunt	VP shunt revision	0.18 mg/kg/hr	142	4	2.38	8	N	N
V	2	Y, Hemodialysis	ARPKD s/p nephrectomies, s/p liver transplant	Endoscopic retrograde cholangiopancreatography	0.72 mg/kg/hr	73	0	4.1	9	N	N
V	3	Y, Hemodialysis	VACTERL, DORV, TAPVR s/p heart transplant, mediastinitis	Cardiac catheterization	0.44 mg/kg/hr	48	0	3.77	2	N	N
V	4	Y, Hemodialysis	s/p liver transplant, AVM, GI bleeding	EGD with sclerotherapy	0.73 mg/kg/hr	55	0	1.97	3	N (Preoperative BIPAP at night)	N
V	5	Y, Hemodialysis	Hemolytic uremic syndrome from shiga toxin, type 1 diabetes mellitus, s/p total colectomy	Kidney transplant	0.34 mg/kg/hr	40	4	2.32	20	Y (BIPAP for two days due to hypervolemia)	N
V	8	Y, Hemodialysis	HTN	Tunneled catheter exchange	0.51 mg/kg/hr	32	1	4.28	6	N	N
V	11	Y, Hemodialysis	TGA s/p repair, heart block w/ pacemaker, SVT, hypofibrinogenemia, diaphragm paralysis	Dental restorations and extractions	0.3 mg/kg/hr	104	0	4.14	7	N	N
V	14	Y, Peritoneal dialysis	Nephrotic syndrome, hypertension	Kidney transplant	0.37 mg/kg/hr	47 (roc ggt stopped to sgm)	0	3.76	12	N	N
IV	8 months^a^	N	High output CKD, atrophic kidney, posterior urethral valve, neurogenic bladder, myelomeningocele s/p VP shunt	Diagnostic laparoscopy, vesicostomy	0.34 mg/kg/hr	206	Not recorded	1.85	10	N	N
IV	1	N	ARPKD, HTN, LVSD	G-tube creation, PD catheter placement	0.39 mg/kg/hr	116	Not recorded	2.5	10	N	Unknown (Epi and Albuterol given for bronchodilation; no rash noted)
IV	1	N	Chiari malformation type II, myelomeningocele, respiratory distress of newborn	Port placement	0.32 mg/kg/hr	81	4	2.15	2	N	N
IV	4	Y, Hemodialysis	Poor nutrition, anemia, hypertension	G-tube creation	0.5 mg/kg/hr	78	0	4.34	7	N	N
IV	6	N	Premature birth (34 weeks), posterior urethral valves s/p nephroureterectomy and ureteral reimplantation	Kidney Transplant	0.61 mg/kg/hr	62	1	3.75	20	N	N
IV	10	N	Posterior urethral valves, hypertension	Kidney transplant	0.34 mg/kg/hr	47	1	3.94	44	N	N
IV	13	N	Treatment resistant Crohn’s, tubulointerstitial nephritis	Dental restorations and extractions	0.21 mg/kg/hr	164	4	2	13	N	N
IV	16	N	Williams syndrome, hypertension, VSD	Renal angiogram	0.53 mg/kg/hr	53	4	2.08	6	N	N
IV	16	N	AKI on CKD due to obstructive uropathy and untreated pyelonephritis, autism	Cystoscopy, suprapubic catheter placement	0.26 mg/kg/hr	47	4	3.12	5	N	N

*Abbreviations*: *CKD* chronic kidney disease, *VP* ventriculoperitoneal, *ARPKD* autosomal recessive polycystic kidney disease, *VACTERL* vertebral defects, anal atresia, tracheoesophageal fistula, renal and limb abnormalities, *DORV* double outlet right ventricle, *TAPVR* total anomalous pulmonary venous return, *AVM* arteriovenous malformation, *EGD* esophagogastroduodenoscopy, *HTN* hypertension, *TGA* transposition of the great arteries, *SVT* supraventricular tachycardia, *LVSD* left ventricular systolic dysfunction, *VSD* ventricular septal defect, *AKI* acute kidney injury

^a^Same patient receiving sugammadex at two different ages

One patient was treated for bronchospasm with epinephrine and albuterol in the operating room after receiving sugammadex. The bronchospasm was brief, resolving quickly after appropriate treatment with no further sequalae. The reaction could have been attributable to positioning of the endotracheal tube or other medications but several factors suggest this patient was predisposed to increased airway reactivity. The anesthesia preoperative history and physical stated that the patient had an upper respiratory infection within the last 4 weeks and had active symptoms of clear rhinorrhea and congestion but clear auscultation of bilateral lungs. The patient tested positive for rhinovirius/enterovirus and negative for COVID-19 three days prior. The anesthesia team proceeded with the case given the urgency of peritoneal dialysis catheter placement for hyperkalemia and newly diagnosed CKD. The timing of bronchospasm so close to sugammadex administration calls into question the possibility of an anaphylactic reaction. Although bronchospasm can be a sign of anaphylaxis, other expected findings such as cardiovascular derangements (hypotension, tachycardia, and shock) or cutaneous manifestations (hives, flushing, and edema) were not present. Anaphylaxis also tends to require lengthier treatment, multiple forms of medication therapy (antihistamines, steroids, crystalloid, bronchodilators, pressors), and even placement of an invasive monitor, to resolve the acute manifestations. The absence of these findings makes anaphylaxis unlikely but it cannot definitively be ruled out without appropriate diagnostic labwork.

## Discussion

Our case series has several important findings. Despite manufacturer and Food and Drug Administration (FDA) of not recommending sugammadex use in severe renal impairment, we found that it was being clinically utilized amongst pediatric anesthesia providers. Notably, there were no instances of deferred extubation, unplanned ICU admission, or need for supplemental oxygen after tracheal extubation, even in a patient with pre-existing noninvasive ventilation need who received a reduced dose of sugammadex. One patient required noninvasive ventilation on POD2 after undergoing a living donor renal transplant. Several findings provide validity that the increased respiratory requirement was primarily due to hypervolemia after multiple fluid rounds of fluid administration. Another had bronchospasm during emergence from anesthesia in the setting of upper respiratory symptoms and testing positive for rhinovirus/enterovirus. The event was quickly and effectively treated with no sequalae. Isolated bronchospasm during emergence from anesthesia is not an uncommon occurrence in pediatric patients and this patient was predisposed to increased airway reactivity and perioperative respiratory complications given the active infection. The absence of several clinical signs and therapies typically found in cases of anaphylaxis makes this an unlikely explanation for the bronchospasm. Thus, based on detailed review of the clinical circumstances surrounding each of these events, we concluded that no adverse events identified were definitively attributable to the administration of sugammadex in pediatric patients with stage IV or V CKD. It should be noted, however, that without quantitative neuromonitoring demonstrating complete recovery of TOF, the possibility of residual neuromuscular weakness cannot be ruled out, even if not clinically present.

Sugammadex and the sugammadex-NMBA complex are renally excreted unchanged. Currently, the FDA does not recommend the use of sugammadex in end stage kidney disease due to prolonged plasma half-life and possible dissociation of sugammadex-NMBA complex in the setting of decreased renal clearance, resulting in free rocuronium that may recurarize. Our study builds upon prior pharmacokinetic studies demonstrating no clinical evidence of residual weakness or recurrence of neuromuscular blockade in end-stage renal patients [7, 8, 12, 13]. The sugammadex-NMBA complex could dissociate theoretically, however, the likelihood may be low given the high association constant (Ka 10^7^ M^−1^) and stability of the complex’s chemical composition [14]. Even when dissociation occurs, rapid re-association may occur due to the strong binding affinity of these molecules, which may make it difficult to identify recurrent neuromuscular blockade, if it occurs at all.

Residual neuromuscular blockade may remain in up to 5% of patients receiving sugammadex [15, 16]. Interestingly, the rocuronium-sugammadex complex was detectable in plasma at day 7 after administration in 6 renally impaired patients in a study by Panhuizen et al., furthering the concern that there remains prolonged exposure of the rocuronium-sugammadex complex in this population [13]. The potential effects of this in the neonatal population requires special attention. Neonates have a large volume of distribution with an extracellular fluid compartment comprising up to 80–90% of the whole body. Elimination can be longer in neonates and recovery from neuromuscular blockade slower, especially if multiple doses are administered. This, combined with an immature neuromuscular junction and lower plasma concentration required to produce paralysis, necessitates careful and patient-specific consideration of risk and benefit when utilizing sugammadex in these populations. Sugammadex administration in this population is not yet approved and further highlights the need for appropriate monitoring of depth of neuromuscular blockade for accurate dosing of NMBA antagonism.

There are multiple limitations in this study. The use of sugammadex in patients with CKD stage IV and V is considered “off-label” and not recommended by the manufacturer and FDA. Since the introduction of sugammadex into our formulary in November 2016, it has been widely utilized [17]. The single center nature of this study is another limitation, including data collection from a single center, small sample size, and risk of non-detection when using ICD coding for identification of subjects [18]. Importantly, recurarization could not definitively be identified due to a lack of quantitative neuromonitoring at our institution. Without the use of quantitative monitoring, the role of sugammadex and residual neuromuscular blockade cannot be dismissed, even if not clinically evident. Given the retrospective review, factors such as type of neuromonitoring device, type of muscle monitored, and presence of fade were not controlled, which challenges the accuracy of sugammadex dosing. Variability in documentation of TOF, timing of drug administration, and other intraoperative events is an inherent limitation of this study. Additionally, no comparisons could be made to be to an age and gender matched historical cohort because of the small sample size. This was a case series designed to describe a range of safety outcomes in pediatric patiens with CKD stage IV and V receiving sugammadex; including a matched cohort would have altered the study design. Although our findings suggest that sugammadex may have a role for neuromuscular reversal in patients with pediatric severe renal impairment, further studies are warranted to elucidate its safety and efficacy in pediatric patients, including the neonatal population who is especially vulnerable given its unique pharmacokinetics.

## Conclusion

In this case series at a quaternary referral medical center, we did not detect adverse effects directly attributable to administration of sugammadex in patients with severe renal impairment. The use of sugammadex in patients with stage IV and V kidney disease requires careful consideration of risks and benefits. Our findings suggest the possibility that sugammadex may have a role for neuromuscular reversal in this population. Routine use of monitoring should be exercised for appropriate dosing of NMBA and NMBA antagonist to appropriately monitor for depth of muscle relaxation and avoid complications related to residual neuromuscular blockade.

## Data Availability

The data (excluding patient identifiable information) that support the findings in this study are available upon reasonable request from the corresponding author after execution of a Data Use Agreement.

## Abbreviations

NMBA: Neuromuscular blocking agents
ICU: Intensive care unit
GFR: Glomerular filtration rate
ESKD: End Stage Kidney Disease
IRB: Institutional Review Board
EQUATOR: Enhancing the QUAlity and Transparency Of health Research
CARE: Case Reports
eGFR: Estimated glomerular filtration rate
CKD: Chronic kidney disease
ICD: International Classification of Diseases
TOF: Train of four
FDA: Food and Drug Administration
RVSP: Right ventricular systolic pressure
